# Unsettling experiences: A qualitative inquiry into young peoples’ narratives of diagnosis for common skin conditions in the United Kingdom

**DOI:** 10.3389/fpsyg.2022.968012

**Published:** 2022-09-15

**Authors:** Abigail McNiven, Sara Ryan

**Affiliations:** ^1^Nuffield Department of Primary Care Health Sciences, University of Oxford, Oxford, United Kingdom; ^2^Department of Social Care and Social Work, Manchester Metropolitan University, Manchester, North West England, United Kingdom

**Keywords:** adolescence, health, qualitative, diagnosis, experiences

## Abstract

Skin conditions such as eczema and psoriasis are relatively prevalent health concerns in children, adolescents and young adults. Experiences of these dermatology diagnoses in adolescence have hitherto not been the focus of research, perhaps owing to assumptions that these diagnoses are not particularly impactful or intricate processes, events or labels. We draw on a thematic secondary analysis of in-depth interviews with 42 adolescents and young people living in the United Kingdom and, influenced by the sociologies of diagnosis and time, highlight the psychological, emotional, social and temporal complexities involved in their diagnosis experiences. Firstly, we describe how participants remembered, re- and co-constructed their diagnosis experiences during the interview. Secondly, we explore the pace and rhythm of diagnosis, including mis-diagnoses, highlighting the jarring potential for adolescents on being diagnosed, even for conditions typically deemed minor. Thirdly, we consider the ways in which these diagnoses have the capacity to reformulate notions of past, present and future, including projecting into imagined futures and reinterpreting past bodily sensations. Finally, we examine how memories about and the meaning of diagnosis are revisited, revised and potentially replaced as a child or adolescent grows older, and increases their management of their condition and encounters with healthcare professionals. In unsettling an assumption that diagnosis experiences for adolescents of common skin conditions is unproblematic or straightforward, our qualitative analysis critically engages with and contribute to tenets of health research that are of interest to quantitative and qualitative researchers, clinicians and patients.

## Introduction

### Common skin conditions and adolescence

Eczema and psoriasis are relatively common skin conditions, with a significant proportion of patients being children, adolescents and young people (Bronckers et al., 2015; Nutten, 2015). Both dermatological conditions are typically associated with a discoloured or otherwise rash-like appearance on the skin and itchiness, although there are important differences in terms of additional symptoms, the underlying causes and mechanisms of the conditions, treatments and management implications, and wider health considerations. Although there are age-related patterns of typical onset and duration, both eczema and psoriasis can affect anyone at any point in their lives and can be long-term (albeit episodic) conditions (Abuabara et al., 2018). Eczema, for example, often affects babies and young children, and there is a commonplace belief that people ‘grow out of’ it when they get older. Psoriasis, on the other hand, often has onset during adolescence and young adulthood; whilst young children can be symptomatic, it is relatively rare.

For people of any age living with skin conditions such as eczema and psoriasis, there can be profound personal and wellbeing impacts (Absolon et al., 1997; Gupta and Gupta, 1998; Carroll et al., 2005; Lewis-Jones, 2006; Basra and Finlay, 2007). There has been a growth in interest in the experiences of affected children, adolescents and young adults as a subgroup (e.g., Fox et al., 2007; Golics et al., 2009; Griffiths et al., 2011; Magin, 2013; van Geel et al., 2016; Ghio et al., 2019, 2020; de Vere Hunt et al., 2020, 2021). This consideration is in recognition that adolescence entails a range of physiological (including neurological), psychological and social changes. Many aspects of life can be affected by developing or continuing to have a skin condition, for example by negatively compounding and complicating adolescents’ experiences with self-esteem and body image, the significance and influence of peer relationships, sexuality and sexual relationships, changes in the dynamics of the family, and independence in domains of life (including health management). Health and illness experiences at this age may subsequently play an important and formative role in shaping future health help-seeking behaviours and outcomes (Angold et al., 1998; Patel et al., 2007; Sawyer et al., 2012; Viner et al., 2012), with different views about the mechanisms through which this may occur (Raphael, 2013).

There has been growing recognition of the specific informational and support needs of adolescents with health conditions, including common skin conditions, with much of the research focus on addressing these needs in the context of individuals having a condition and the ongoing impact (Stinson et al., 2013; Kirk and Milnes, 2016). Where focus has been on the role and impact of diagnosis, these usually involve conditions in which absent or delayed diagnosis and subsequent treatment has significant morbidity and mortality risks, including around newborn screening (Berg et al., 2014; Gambling and Long, 2019; Ke et al., 2019). In contrast, research on common skin conditions has largely focused on experiences of *living with* the condition and the impact on quality of life – with diagnosis seemingly a prerequisite but otherwise unimportant event of the past (Fox et al., 2007; Ablett and Thompson, 2016). This may stem from assumptions that the diagnostic process is clinically straightforward for eczema and psoriasis diagnoses, and that, as common ‘minor’ conditions, the impact of diagnosis is not one of shock or surprise (Green, 2010; Schofield et al., 2011).

### Diagnoses, as processes and products, in illness narratives

From a clinical and pragmatic perspective, diagnosis serves a purpose of naming and making sense of a constellation of symptoms, signs and markers, and can be an important component in laying a map for management and treatment options. A diagnosis can be made through a range of human (professional) and technological forms of assessment, and marks a high degree of certainty about symptoms and markers – even if the underpinning evidence-base contains questions regarding the nature and mechanism of a condition, the best courses of treatments, and accurate prognosis. In the sociology of diagnosis literature (e.g., Jutel, 2011, 2014; Jutel and Dew, 2014), further consideration is given to the socially constructed nature of organising bodily experiences into a medical framework, and the myriad of psychological, emotional and social meanings and consequences that this can have for patients. Diagnosis is unpacked to consider both the process (of diagnosing) and the product (a diagnostic label), and that these two related but not synonymous aspects can have intentional and unintentional impacts beyond having a simple clinical purpose.

One of the things that diagnosis can ‘do’ is to structure accounts of time with regards to a health condition or event. A linear narrative, although variable for different types of health conditions and events as well as structures of health services, might typically begin with the development of symptoms, move to consulting a health professional and having investigations, then receiving a diagnosis, and beginning a course of treatment or management (Brossard and Carpentier, 2016). Illness accounts may therefore feature diagnosis as part of the backstory to the present and identify diagnosis (as a process, event and/or label) to be a ‘turning point’ in the biographical construction (Undeland and Malterud, 2007; Germeni et al., 2018). Implicit within this is the recognition that diagnosis is not necessarily a neutral event or action; receiving and having a diagnostic label can shape an individual’s orientation to and activities within their lives, potentially invoking re-evaluation and revision (for example, of expectations for the future). Bury's (1982) concept of ‘biographical disruption’, although not solely concerned with diagnosis, implies that *becoming ill* marks a distinction in the life of a person/patient: the way they think and feel about their life has changed in an unexpected manner. Whilst symptoms and the experience of *becoming* or *being* ill can go unnamed and undiagnosed (officially or otherwise), the process of seeking a diagnosis and the label of having a diagnosis are cornerstones in much of modern medicine.

The literature capturing illness experiences in terms of the onset, diagnosis, and continuation of illness has expanded, with exploration of a range of different health conditions/events and patient groups. The concept of ‘biographical disruption’ (Bury, 1982) has been challenged and nuanced through the lens of particular health conditions and experiences, with the emergence of concepts such as biographical- ‘repair’, ‘continuation’, and ‘abruption’. Locock et al. (2009) outline biographical repair as seeking to “restore normality and control” (p. 1050), and biographical continuation, drawing on Williams (2000), as when illness or ill health events are “absorbed and accepted within one’s existing trajectory as biographically anticipated” (p. 1045). With their focus on terminal illness, Locock et al. (2009) offer the concept of biographical abruption as embodying a sense of “a ‘death sentence’, that life was in effect already over” (p. 1047). In addition there is recognition that having a diagnosed condition (symptoms and a diagnostic label) may have little to no impact for a person, particularly if symptoms are deemed relatively insignificant, ‘normal’ in their social circle, as ‘always’ having been there, or contextualised with experiencing other hardships (Pound et al., 1998; Faircloth et al., 2004; Harris, 2009; Monaghan and Gabe, 2015). The biographical impact of a (diagnosed) condition has been explored in the context of Motor Neurone Disease (Locock et al., 2009), hepatitis C (Harris, 2009) and various cancers (Schaepe, 2011); with the exception of skin cancer (Winterbottom and Harcourt, 2004), dermatological conditions have been largely absent to date. Following critiques that earlier literature focused only on adult experiences, subsequent consideration has attended to the experiences of children, teenagers and young adults (e.g., Williams, 2000; Williams et al., 2009; Bray et al., 2014; Monaghan and Gabe, 2015).

Our qualitative paper adds to understandings of the impact of skin conditions, adolescent health, and the sociologies of diagnosis, time/temporality and illness, by exploring how young people with eczema and psoriasis describe and account for their experiences of diagnosis – as processes and labels, with psychological, emotional, social and temporal impacts.

## Materials and methods

A secondary analysis was undertaken of in-depth interviews with 42 young people aged between 16 and 24 years from England, who shared their experiences of having eczema or psoriasis. The original qualitative study was conducted with the aim of exploring the information and support needs of young people with common skin conditions. A total of 88 participants with acne, eczema, psoriasis and/or alopecia were interviewed. Participants were living in England and ranged in age from 14 to 24 years. The interviews were conducted between October 2014 and December 2015. The original study was approved by Berkshire National Research Ethics Service Committee (South Central) and was funded by National Institute for Health Research under its Research for Patient Benefit scheme (PB-PG-0213-30006).

The authors of this paper (AM, SR) are two of the original researchers, and the impetus for the secondary analysis was in part influenced by the first author’s interest in diagnosis as informed by their work on another health condition from a different vantage [the role of diagnostic processes and labels for endometriosis from the perspective of NHS General Practitioners (Dixon et al., 2021)]. The question our secondary analysis addressed was ‘how do young people experience and make sense of diagnoses in the context of eczema and psoriasis?’ Secondary analysis involves analysing data generated for a different process (Heaton, 2004). While there is some contention over the meaning of secondary as opposed to primary analysis, and the extent to which the two approaches may differ (see, for example, Hammersley, 2010), we have asked a different question of a self-collected dataset (Heaton, 2008) and this has involved undertaking a distinct analysis. As researchers on the original study, we also bring to this secondary analysis a detailed understanding of the design and delivery of the study, including additional context regarding the dataset to aid interpretation, whilst also capitalising on the ability of a secondary analysis to explore previously under-considered topics and new perspectives. Our secondary analysis focuses on two of the four dermatological conditions in the data set (eczema, psoriasis), totaling 42 interviews, and excluded the data from participants whose interviews were solely about experiences of acne or alopecia.^1^

The original study had a ‘maximum variation sample’ design (Coyne, 1997) to include a range of demographic factors and contexts which might impact on young people’s experiences of skin conditions. In addition to the type of dermatological condition, these factors included: age, gender, ethnicity, social class, geographic location and study/employment status (see Table 1 for an overview of key demographic characteristics). Participants were recruited through healthcare settings (via posters and fliers in waiting rooms, recruitment packs distributed by health professionals in primary and secondary care), support organisations (charities, mailing lists, discussion forums, social media), universities, colleges and schools, and through social media platforms such as Facebook and Twitter. Detailed information sheets were provided and participants were given opportunities to discuss their participation throughout the study. Participants gave written consent and, for those under the age of 16, parental or guardian consent was also required.

**Table 1:** Demographic characteristics of the secondary analysis sample (n.42).

	Eczema	Psoriasis	Total
*Number of participants*	24	18	42
**Age (years)**			
14–17	3	6	9
18–21	13	5	18
22–24	8	7	15
**Gender**			
Female	17	11	28
Male	7	7	14
**Ethnicity**			
White British	13	15	28
Asian or British Asian	8	3	11
White European	1	0	1
Black Caribbean and White British	1	0	1
Arab and White British	1	0	1

All but one interview were conducted by the first author (AM) in participants’ homes or, if they preferred, in alternative meeting spaces such as community centres. Participants could choose to be interviewed on their own or with others present, including parents, guardians or a friend. Of the 42 participants included in the secondary analysis, only one chose to have a companion present throughout the interview; however, as will be highlighted later, family and friends sometimes joined the interview fleetingly. AM remained reflexive about her position as a (then) young, white, female researcher with a personal history of skin conditions (mild eczema, facial acne) sometimes evident, and SR provided supervision and support throughout the study.

The interviews were semi-structured, with an opening question inviting participants to talk freely about their experiences of a skin condition. Follow-up questions based on a topic guide ensured that areas not yet mentioned but present in existing literature or highlighted in previous interviews in the study were raised for discussion. Topics covered included symptoms, seeing healthcare professionals, using treatments, impacts on emotions and identity, impacts on friendships and relationships, financial costs, experiences across the duration of having the condition (including different contexts, e.g., at school, college, university, jobs) through to the present day, and looking ahead to the future. Interviewing continued until data saturation was reached, as marked by the point when the research team felt that no new perspectives or differences of opinion were presented in subsequent interviews, with the dataset concluding at 88 interviews (Hennink and Kaiser, 2021).

The original dataset was coded by first author AM. The first few transcripts were independently coded by second author SR. The coding was compared, inconsistencies discussed and resolved. A working coding framework was developed for the remaining data. For the secondary analysis, NVivo software was again used to organise the two subsets from the original dataset. Data were re-coded by first author AM using a coding framework based on interview content and relevant literature.This was refined throughout the process in discussion with SR as our analysis focused on the meaning the diagnosis held for participants and the ways in which they made sense of it. Thematic coding reports were then analysed more conceptually for links and connections across the data as well as the identification of outlier examples. Repeated questions were asked of the findings with discussion between both authors to ensure a robust and iterative engagement with the data. All participant names featured in the paper are pseudonyms.

## Results

### Reconstructing and co-constructing diagnosis experiences

Some participants could remember firsthand their experiences of developing symptoms, consulting for support, and getting a diagnosis for their eczema or psoriasis. Whilst some could not recall the in-depth detail and content, or step-by-step process, of *how* they came to be diagnosed and the information that accompanied the diagnosis, their emotional responses were often vivid in their memories. Lara (age 16, female) was 7 years old when she was referred to a dermatologist and diagnosed with psoriasis during an appointment:

*I didn’t even want to go to the hospital, I just wanted to go to school. And then I sat there and I remember my mum trying to explain to me. I knew that it was something to do with my skin but I didn’t know what it was.*

The emotional responses to diagnosis – which could include confusion, shock, surprise, indifference or relief to have a medical label – could be exacerbated if the circumstances of diagnosis were unexpected and outside of the typical pattern of consulting healthcare professionals about particular dermatological symptoms. Anthony (age 17, male) was unexpectedly diagnosed when he was 10 years old and hospitalised with a serious blood infection. Although Anthony had itchy and dry skin for some time, he had not seen this as a problem or indicative of a skin condition until an emergency care doctor “*said, ‘This [infection] is due to your eczema,’ and I was-, obviously-, I didn’t know I had eczema at that time until the doctor said*.” The diagnosis was unexpected and the seriousness of his infection added to his sense of shock. Anthony said he *“didn’t know how to feel*” at the time but with hindsight, and from the vantage point of the interview, the diagnosis marked a change in his life and perception of himself: “*I used to be a really healthy kid.”*

Not all participants could remember their symptom onset or being diagnosed, often because they were young infants at the time. As such, some felt that there had not been a time in their lives ‘before’ they had a skin condition: “*it’s always been part of me. I’ve never experienced having no eczema, so for me this is normal*” (Jo, age 17, female). In this formulation of always having had eczema, there is not an illness narrative of disruption with onset. However, whilst participants may view themselves and the presence of their condition as always existing simultaneously, implicit in the naming of the condition and speaking of management/treatments is that a diagnosis has taken place at some point. Again, often as very young infants, this was not something participants could necessarily speak to from their firsthand experience – yet they drew upon various ways to reconstruct the process of seeking and receiving diagnosis, namely through their parents/guardians and other family members’ memories.

There were different ways that participants reconstructed the order of events that preceded their memory, including experiences of diagnosis. Some had heard about the processes from their parents or wider family, and absorbed this history into their telling of events. For others, the forthcoming interview was itself a prompt to ask family members for information about the timings and processes, such as when and how their skin condition had first been suspected and named. Some participants contacted the researcher in the hours, days or weeks after the interview to fill in some ‘gaps’, based on conversations they subsequently had with their parents and wider family. Additionally, parents were sometimes present, brought into or invited themselves into the interviews to give information at key points, highlighting the co-constructed nature of qualitative research encounters and data production.

These forms of parental/guardian contributions participated in the production of the participant’s narrative in the interviews and afterwards, providing additional context for sense-making of the data. During the interview, for example, Bethany (age 22, female) described her earliest memory of eczema as going to nursery when she was a few years old and becoming “*aware of others [peers] and what they do and what they can eat*,” including memories of being prohibited from activities related to her triggers (e.g., petting animals). After the interview, Bethany asked her parents to tell her more about the earlier experiences, moving a step further back into her history beyond what she herself could recall. She explained that the ‘start’ of her eczema was when she was a few months old, at the point of weaning (“*[my] parents knew when I started on solids and started to get rashes in reaction to certain foods*”), and from there her parents consulted healthcare professionals, she was diagnosed, and treatment and management ensued. This part of Bethany’s history – including the occurrence and significance of diagnosis – was not part of her firsthand memory but re-and co-constructed via her parents participating in the production of research data.

### The pace and rhythm of diagnosis

In addition to the variation in terms of age at diagnosis, the process of seeking and receiving a diagnosis took different amounts of time and was experienced in different ways by the children, adolescents and young adults in the study. Sometimes diagnosis was rapid in pace and with minimal investigation: a person has some symptoms, they promptly attend an appointment with their GP, they describe and/or show the symptoms, and a diagnosis is made. This type of swift process was most often described about eczema and diagnosis was made in primary care. For others, whilst the process of being diagnosed once inside the consultation room may take just seconds or minutes, the lead-up to deciding to see a health professional and book an appointment may have taken much longer (months, weeks, even years). There were a variety of ways that diagnosis was experienced in terms of pace and rhythm; the ‘standard model’ of diagnosis (Brossard and Carpentier, 2016) – whereby symptoms are noticed, an appointment is attended, and a diagnostic label is ascribed – was not universal, as noted earlier in Anthony’s abrupt diagnosis in an acute situation of infection after years of itchy skin which was not (yet) deemed to be a symptom.

For some participants, particularly those with psoriasis, the process of seeking a diagnosis was neither quick nor contained, and could involve multiple investigations and tests, referrals to secondary care, and long periods of time where symptoms were not yet explicitly or formally named. Whilst these diagnoses spanned longer periods of time, there were different perceptions of pace and rhythm in these experiences. Participants described how they sometimes waited for periods of time with little to no contact with healthcare professionals and services, whilst other times the time period included multiple consultations, tests, and conversations in quick or otherwise intense succession, including discussions about possible or speculative diagnoses without definitive conclusion. Medical tests were sometimes undertaken to eliminate alternative explanations for the symptoms, with time spent not only attending for tests but also arranging them and waiting for the results. The content of these experiences of seeking a diagnosis contributed to perceptions of pace, in terms of whether the time spent ‘waiting’ for diagnosis moved slowly and felt empty, or conversely felt intensely busy and full of activity.

Based on their age, some children and young people felt that their healthcare professionals were initially hesitant to give them particular diagnoses and focused on eliminating all other options first, which also added to the length of time that these individuals felt ‘in limbo’ about their health situations and management options. This was most often described in relation to psoriasis, whereby it had been diagnosed and managed as something else initially, including as eczema. As noted earlier, there are age-related trends for the onset of psoriasis; for children displaying symptoms consistent with the diagnosis, other explanations were usually considered first and explored with trials of treatment. Lara felt that she was not diagnosed with having psoriasis sooner or quicker because “*it’s really rare for children under the age of 12 to get it, so I got treated as an allergic reaction, and then eczema, and then another allergic reaction*.” As well as being related to age, other aspects deemed medically unusual or unfamiliar to some healthcare professionals could lead to first considering alternative diagnoses. For example, Louise (age 20, female) saw different GPs about her symptoms of an “*itchy vagina*” and it was repeatedly implied that her symptoms were likely to be a sexually transmitted infection, despite multiple tests with negative results. Louise took to looking online and was surprised how quickly she saw references to ‘vulval eczema’ – a diagnosis which, several months later, was confirmed by a specialist.

There were also practical challenges for participants in using healthcare services, stemming from uncertain or revised diagnoses. For example, Daniel (age 15, male) has psoriasis but was initially told he had a fungal skin infection and prescribed topical antifungals by his GP. Although his symptoms did not clear up, it took a while for him to be able to return to his GP as it required him to take time out of school and for his parent to take time off work to attend another appointment. The cost of prescriptions (for young adults eligible to pay) and over-the-counter treatments for an undefined or misdiagnosed condition could add to a sense that the process had been a waste of money as well as of their time. As such, uncertain, revised and misdiagnoses presented challenges for children and some adolescents and young adults, as well as their families, stemming from dependence on parents or guardians to transport and accompany them to see healthcare professionals, and having little or no financial independence to draw on in their healthcare management.

Young people sometimes described feeling ‘let down’ when healthcare professionals did not or were unable to conclusively provide a diagnosis, or when an earlier diagnosis was revised. Frustration was described by Gemma (age 23, female) who saw her GP many times over several weeks and months before being referred to a dermatologist who quickly diagnosed her with psoriasis in the appointment: “*it took 30 seconds*.” However, because of the delays involved in seeking the diagnosis, Gemma had widespread and extremely itchy, cracked and flaky skin at a severity which meant corticosteroid topical treatments would not be suitable and she would have to wait a further 6 weeks before phototherapy commenced, compounding her disappointment that the diagnosis process had been quick in the dermatology appointment but drawn out across months because of other aspects of the healthcare services. The implications of hindsight for young people, including Lara, was a sense that diagnosis could and should have been quicker, and, from the vantage point of having a diagnosis, young people also evaluated their past and future in other ways too.

### The impacts of receiving a diagnosis on perceptions of time

Participant narratives highlighted how diagnosis – as processes, events and labels – could shape views about past, present and future. As such, for some young people, diagnosis marked a biographical disruption in terms of their thoughts and feelings about themselves, and expectations about how other people (especially peers and potential partners) might think about and behave towards them in the future. James (age 22, male) summed this up when he described receiving a diagnosis of psoriasis aged 17, as the point when “*bang, it’s all changed*.” Daniel also described how upsetting diagnosis could be (“*like a dagger in the heart*”) and especially when accompanied with information such as the “*possibility that I could have it for the rest of my life*.” Returning to the emotional impacts of diagnosis for Lara, she recalled how:

*I just kind of sat there [in the appointment], like this is going to entail on my whole life, I could have it for the rest of it, and people are going to think it’s ugly and horrible, and I’m going to lose so many people because of it.*

The concerns that Lara described having at the point of diagnosis, however, cannot be easily separated from her experiences in the following months and years which did include friendships ending and being bullied about her psoriasis. From the vantage of the interview, the fact she had indeed experienced social, emotional and psychological losses reinforced diagnosis as a significant (negative) turning point, again highlighting the complexity of qualitative research and narrative interviewing as retrospective.

However, this sense of biographical disruption was not felt by all participants. Some did not describe receiving a diagnosis as important in terms of affecting how they thought or felt about themselves. As mentioned earlier, some young people who had eczema since they were a baby or toddler deemed that their skin condition had ‘always’ been there and part of them. There were also some participants who developed their condition at a later age but for whom it was not considered particularly significant or impactful in their lives. When asked about experiences of ‘diagnosis’ in his interview, Matt (age 20, male) thought it was “*quite a strong word*” and not fitting in his case of having “*a touch of psoriasis, only in certain places*,” rather than “*a serious, horrible illness*.” As such, the symptoms of the condition, and the process and label of diagnosis, were both relatively insignificant for Matt.

Asides from the personal, emotional and psychological impacts on individuals from a diagnosis – including the potential, or not, for biographical disruption – young people often found that a diagnostic label had a useful social function in the present. Being able to give a medical name for a condition could help young people explain their symptoms and reassure, or minimise unwanted comments from, friends and family as well as wider peers, strangers and/or colleagues. John (age 21, male) found having the label ‘psoriasis’ helpful to explain his “*red, angry, blotchy*” skin to friends and family who had often asked questions or made comments:

*Once I knew it was psoriasis, I could say, ‘Oh, got this, don’t know how long it’ll be there for, hopefully it’ll go away’ […] [rather than] ‘I don’t know what it is, it’s just something that’s there’.*

For some participants, having a diagnostic label helped distance them from adverse and stigmatising connotations that might otherwise be associated with their visible symptoms, certain behaviours and sensorial aspects of treatments (including the smell and feel of topical treatments). This included others’ concerns about contagion and questions over the person’s hygiene, which might underpin upsetting comments and behaviors which, as Lara experienced, included bullying and friendships ending. At a stage in their lives where the approval of peers and potentially relationships are paramount, a diagnostic label could be a welcome tool for adolescents in trying to avoid various types of social exclusion and rejection.

In addition to the ways that views about the present and imagined futures were affected following diagnosis, some young people also described looking back at the past in a new light. This included (re)interpreting bodily sensations that had previously been contained as unimportant or otherwise temporary, including itchy skin for Anthony which, through naming as eczema, became symptoms of a condition he had, has and is likely to continue having in the future. As such, whilst receiving a diagnosis could cause individuals to revisit their thinking about their experiences, in the fourth and final theme, we explore how this is an ongoing process over time.

### Growing independence and re-diagnosis

Memories about and the meaning of diagnosis can change as time passes and a child or teenager grows older, has increased independence, learns more about their health, and takes on a larger share of responsibilty for the management of their skin condition or health and life in general. This was especially pertinent to those whose diagnoses had taken place when they were babies or young infants and who had been managing a skin condition for many years, but who could not recall firsthand the experience of being diagnosed. In addition to the information given at the point of diagnosis, parents or guardians had usually been the primary recipient of information from healthcare professionals about the skin condition across many years – as well as having the overarching responsibility for management and treatments. This was the case for Zoe (age 20, female), who had eczema since she was an infant but “*don’t really understand what was wrong with my skin*” and *“just lived through it without any information*.” The point at which the parental-doctor dynamic becomes less appropriate and necessary, and a transition takes place, should be informed by the ability of the child or adolescent to understand information and take on (greater) responsibility for treatments. However, for some young people, the dynamic was difficult to disrupt and could be enduring even into early adulthood.

As the young people grew older, the feeling of being a bystander in their own medical care could leave them feeling marginalised, confused and frustrated – including lacking information that might have been delivered alongside diagnosis and in additional consultations. In Zoe’s experience, healthcare professionals, including GPs, “*don’t retell you the things they expect you to know*” and “*just believe that as a child you understood and you’ve carried on*.” Zoe thought that health professionals perhaps assume that parents relay information about the diagnosis, in addition to ongoing treatment regimens, on to the child as they grew up, but cautioned that this does not always happen or it could consist of partial and sometimes inaccurate information. As such, children, adolescents and some young adults felt that they lacked information about the causes and triggers for their condition, or how treatments worked, as they grew up. For some, participating in the interview led them to realise that they had gaps in their current knowledge and unanswered questions which no-one (healthcare professional or parent) had hitherto explained or done so in a way that they understood.

Other young people had acquired information which would normally accompany diagnosis or early consultations when they started having more independence with their health and attended medical appointments without their parents or guardians. This was the case for Natalie (age 19, female) who had had eczema since she was a baby; after moving away to university, she struggled with disrupted sleep because of the itchiness of her skin and so she booked an appointment. Here, the practice nurse drew a diagram of the skin and the mechanisms of eczema: “*It’s the first time anyone’s ever explained why I have eczema, what causes eczema and the best ways to treat it […] It took 19 years, but someone finally explained.”* Such an example highlights how young people’s encounters with healthcare professionals at an older age could be experiences of revisiting, revising and potentially replacing or filling in gaps of their former understandings of the diagnostic label, constituting a type of re-diagnosis.

Just as some young people found it distressing to receive a diagnosis, those who had types of re-diagnosis could also feel shocked or surprised and experience it as a biographic disruption which affected their perception of the past, present and future. Indeed for some young people, having lived with and managed a diagnosed condition for years could make it even more surprising to learn something that they felt they should have known all along. Kirsty (age 17, female) felt that the information her parents had been given when she was diagnosed with psoriasis as a child was lacking and, as a result, they were left “*to find out a lot for ourselves*,” for example through online research. At a recent appointment with her doctor, she was shocked to hear about the increased risks of cardiovascular disease and for it to be raised flippantly, implying that she should already know or would otherwise not find it frightening to hear:

*I remember the doctor saying, “Oh when you’re 40, let your GP know that you have got psoriasis.” I was a bit shocked by that. […] I found it quite crude. She was like “Oh let them know.” She kind of said it as a joke and I was like, “Hang on a second, I did not realise- [that psoriasis could affect heart health].”*

Rather than diagnosis being a static and completed event from the past, young people grappled with these kinds of ‘re-diagnosis’ as reverberations when they grew older and information emerged in medical appointments and/or through online research. As such, whilst the original medical diagnosis may not have been experienced as a turning point, including for infants who have no firsthand memory of the process and event, these types of events in later years and with increased health responsibility could add to, revise or replace previous experiences, meanings and knowledge associated with diagnosis.

## Discussion

A substantial body of medical sociology literature has considered the onset of a health condition or event as having the capacity to shape a person’s approach to their life and health, including in terms of their perspective on the future, with diagnosis often an implicit apriori component (Bury, 1991; Faircloth et al., 2004; Demain et al., 2015). For example, diagnosis sometimes features as a ‘turning point’ in people’s stories of health and illness as a mechanism for distinguishing time in particular ways, such as ‘before’ and ‘since then’. Our paper argues for viewing diagnoses, as processes and products, as experiences worthy of academic consideration; in other words, approaching diagnosis as a topic in its own right and not only the ‘start’ of stories about living with a condition (Jutel and Nettleton, 2011). We reiterate how diagnoses can mark important changes in how people view themselves and their futures, and how they interact with others (Jutel and Dew, 2014), but not always and in all cases for the same condition. By considering young people with common skin conditions, the paper expands the contexts in which illness experiences (and diagnoses specifically) have been considered in the academic literature, and argues that being diagnosed with a common skin condition, contrary to popular beliefs that these are not ‘serious’ illnesses, can be upsetting and confusing for young people.

The distress described by some in response to the processes and product of diagnosis suggests that childhood and adolescence can be a fragile time to develop, be labelled with, and manage a skin condition such as eczema or psoriasis. These conditions are not typically deemed to be life-threatening, though we note complications such as Anthony’s experience of a blood infection can be, yet they are life-impacting. Existing literature highlights the impact on aspects such as self-esteem and mental health more broadly (Yeo and Sawyer, 2005; Shaw et al., 2019). In addition to the impact of the condition itself, our data suggest there are a range of ways that young people can be affected by the processes, events and labels of these diagnoses. However, not all participants described the processes of seeking and receiving diagnosis as significant to them, nor as constituting a disruption to their lives – either previously, currently or in their imagined futures. In some cases, a sense of biographical continuity and endurance—of always having had the condition or minimal disruption—meant that it was deemed part-and-parcel of their lives, and not remarkable. For some, being very young and having little direct memory of the diagnosis meant that the occurrence had little temporal potency in delineating ‘before’ and ‘after’. As such, this paper contributes to challenging the ways in which, as Brossard and Carpentier (2016, p. 2) have written in the context of dementia, “diagnosis continues to be treated as a self-evident turning point” in patient and/or research participant lives, when in fact these may not always be.

As Goodwin and McConnell (2014) argue, diagnosis is often not simply a ‘moment’ but a process which extends over time, and our analysis has further explored the ways in which time and temporality can be involved in young peoples’ experiences. This has included exploring the pace, rhythm and reverberations of diagnosis, as well as considering chronological age at various stages of reconstructing, remembering, and revising diagnosis over time and growing older. The length of time before a diagnosis was given following onset of symptoms and/or first consultation with a health professional could take days, weeks and months – for some, seeking a diagnosis became a burdensome task and could feel simultaneously protracted and yet, when faced with a given diagnostic label, abrupt and shocking. For some participants, there was a sense that the diagnosis *could* and *should* be quicker, and therefore that their experience of diagnosis had been slower than necessary. The lack of immediacy of diagnosis, or at least a sense that these were often not made in a timely enough manner, left some young people feeling unsupported and frustrated.

Receiving a diagnosis could then end a period of uncertainty for the young person about the meaning of their symptoms, potentially offering reassurance about the next steps of management and treatment. Having a medical name for their condition could be a useful label for explaining visible symptoms and potentially mitigate against stigma that, for example, they had a potentially contagious disease or were ‘dirty’. However, some participants—such as Lara—described having an immediate sense on receiving a diagnostic label that their peers would nonetheless stigmatise and alienate them. For some young people, the experience of ‘gaining’ a diagnosis subsequently figured more as a personal, emotional, psychological and social injury in some ways, as Daniel’s reference to diagnosis being “*a dagger in the heart*” strikingly conveyed. Biographical disruption was expressed about receiving (as well as having) a diagnosis, when young people anticipated there subsequently being unwanted changes and various types of losses in their future pertaining to their skin condition.

We suggest that part of the distress felt by some young people on receiving a diagnosis for eczema or psoriasis pertains to the way in which their actual experiences did not marry up with their socialised understandings of expectations about medical professionals and medical processes. For some, this was their first experience of consulting for a health concern, with varying degrees of parental/guardian involvement, and diagnosis was part of their expectations meeting reality; their expectations about how a diagnosis would be made, how quickly, and what would happen next sometimes jarred with the reality they encountered. One area where there were added complications to expectations and realities of diagnosis for young people concerned misdiagnoses. These experiences could be unsettling or even shocking for children, adolescents and young adults, challenging a naivety that they had previously held about the certainty of healthcare professionals, processes, systems, services and knowledge. Not only could experiences of uncertainty and/or misdiagnosis undermine children and adolescents’ beliefs about and confidence in medical processes, professionals and services, but it could also have tangible impacts for them in terms of feeling they had ‘wasted’ time before more appropriate treatment could be started and symptoms better managed.

Partial or unclear communication of information by healthcare professionals and misunderstanding on the part of the patient is a concern across all groups, but our data suggest it may be particularly relevant for children and adolescents in the context of diagnosis owing to their circumstances. The parents of those who were diagnosed when they were very young were primarily the recipients of information, but this content is not necessarily shared clearly with the child as they grow up and may not be repeated by health professionals subsequently seen. Even for those who had reasonable capacity to comprehend the diagnosis at the time, information and explanations may not be directed towards them or communicated in an age-appropriate way. This also highlights the risk of informed healthcare falling between the gaps when a parent or guardian is a proxy holder of the condition-specific information. Some young people diagnosed when very young had experiences of seeing health professionals when they had become more independent in managing their condition, for example when attending appointments without their parents, and being helped to understand the background to their condition. We suggest these experiences constitute a type of re-diagnosis, as time folds back in a sense to allow them to comprehend that which they could not or did not at the time of the official diagnosis. The learning of new information, which could invoke surprise and worry, could add to a sense of disruption for those who felt the diagnosed condition marked (or now marked) a significant change in their lives.

## Conclusion

The focus of this secondary analysis has highlighted some of the complexities surrounding the diagnosis of two common skin conditions – eczema and psoriasis – for children, adolescents and young people, and the ways in which they make sense of this diagnosis in the context of time and over time. Various aspects of time and temporality have been drawn out of the accounts given by young people regarding their diagnoses, highlighting different features deemed important, including: the age circumstances of when diagnosis takes place and their ability to remember or reconstruct it, the pace and rhythm of seeking a diagnosis, feelings on receiving a diagnosis and having a diagnostic label, the impact of diagnosis on perceptions of past, present and future, and the way diagnosis can be revisited and revised as the young person grows older and more independent. Our analysis has also highlighted the interactional layers involved in young peoples’ diagnosis experiences, as well as in the processes of recalling and narrating experiences in the setting of a qualitative interview. We hope our paper will showcase the depth and rich insight that a qualitative exploration can yield on a topic – diagnoses – that is of interest to both quantitative and qualitative researchers, as well as patients and clinicians.

Our findings are in the context of recognition that early encounters with healthcare professionals, particularly those involving growing degrees of independence for health management with age and maturity, can impact future approaches and expectations towards help-seeking and health management. We suggest though that the temporal context is important for understanding why a diagnosis of eczema or psoriasis might be upsetting for children and adolescents, given that these are not widely regarded as particularly serious or shocking diagnoses nor conditions. This includes the fact that it may be the individual’s first or most significant to date reason to engage with healthcare professionals and services, and that their expectations about medical processes and the operations of the healthcare system (in this context, the NHS) may jar with the realities they encounter – including that diagnosis may be slower and more complicated than expected. In addition, it may also be the first time they have had been told they have a long-term health condition, rather than an acute medical event such as the flu or a broken bone, and reconciling the meaning of this in the context of their lives where aspects such as the importance of peer opinions, appearance concerns and growing independence are increasing with the transition of adolescence.

Late childhood, adolescence and young adulthood often constitute periods of significant change, including growing independence around health condition management and help-seeking. The present paper contributes to medical sociological literature and to evidence demonstrating the impacts of skin conditions on young people, recognising them as an important group with particular information and support needs. Looking through a lens of time and temporality has also highlighted possible relevant areas for improvement in health care, such as the need for professionals to manage young peoples’ expectations about the likely duration, pace and rhythm of seeking diagnoses when these conditions are a possibility.

### Study implications

It is important that healthcare professionals, including psychologists, consider that diagnosis experiences for typically regarded minor conditions like eczema and psoriasis can be distressing and troubling, particularly for children and adolescents for whom there are additional confounding factors. We also suggest it may be pertinent to revisit information that would have accompanied diagnosis with adolescents, as they reach various stages of independence in their lives and in their approach to healthcare, in recognition that there may be gaps in understanding.

### Strengths and limitations

A strength of this paper is the use of concepts of time and temporality – including chronological age, as well as qualities like rhythms, pace and reverberations – which offer a productive lens through which to explore narratives of young people with two common skin conditions, eczema and psoriasis. The original study considered a wide range of young people’s experiences, with a large sample size for a qualitative study which offered rich data for our secondary analysis. Nonetheless, a limitation is that other important differences may exist that were not adequately articulated in the data set and therefore not drawn out in our analysis, and our paper has not been able to explore all angles.

## Data availability statement

The original contributions presented in the study are included in the article/supplementary material. The datasets used in the secondary analysis are available from the corresponding author on reasonable request.

## Ethics statement

As a secondary analysis, ethic review and approval was not required for the study on human participant in accordance with the local legislation and institutional requirement. Written informed consent from the participants’ legal guardian/next of kin was not required for the secondary analysis study in accordance with the national legislation and the institutional requirement.

## Author contributions

AM conducted the interviews in the original study. SR provided supervision and support throughout the original study. All authors contributed to the article and approved the submitted version.

## Funding

This project is funded by the National Institute for Health and Care Research (NIHR) under its Research for Patient Benefit (RfPB) Programme (Grant Reference Number PB-PG-0213-30006). The views expressed are those of the author(s) and not necessarily those of the NIHR or the Department of Health and Social Care.

## Conflict of interest

The authors declare that the research was conducted in the absence of any commercial or financial relationships that could be construed as a potential conflict of interest.

## Publisher’s note

All claims expressed in this article are solely those of the authors and do not necessarily represent those of their affiliated organizations, or those of the publisher, the editors and the reviewers. Any product that may be evaluated in this article, or claim that may be made by its manufacturer, is not guaranteed or endorsed by the publisher.

## Author disclaimer

The views expressed are those of the authors, and not necessarily those of the NHS, the NIHR or the Department of Health.

## Footnotes

^1^The dominant symptoms that participants had and consulted healthcare professionals about were deemed important in underpinning their diagnostic experiences, and it was felt that a focused secondary analysis on eczema and psoriasis would be most insightful. Whilst there are important differences in the symptoms of psoriasis and eczema, participants described some similarities – including a discoloured or otherwise rash-like appearance on the skin and itch – compared to the dominant symptoms described for acne (spots/pustules) and alopecia (hair loss). Other study findings from the acne and alopecia datasets have previously been published (McNiven, 2019; de Vere Hunt et al., 2020) which, to varying degrees, highlight aspects of diagnostic experiences for these dermatological conditions in the age group of adolescents and young people. For example, participants with acne often reported ‘knowing’ they had acne (or ‘spots’) without the need for suggestion or confirmation by medical professionals (McNiven, 2019).
